# IDENTIFYING, DEVELOPING, AND IMPLEMENTING CARE PLANS CONSISTENT WITH GOALS, PRIORITIES, AND PREFERENCES

**DOI:** 10.1093/geroni/igad104.0962

**Published:** 2023-12-21

**Authors:** Tonya Roberts, Laci Cornelison, Tetyana Shippee, Mary Lou Ciolfi, Susan Ryan, Penny Cook

**Affiliations:** University of Wisconsin, Madison, Wisconsin, United States; Kansas State University, Manhattan, Kansas, United States; University of Minnesota, Minneapolis, Minnesota, United States; University of Maine Center on Aging, Bangor, Maine, United States; Center for Innovation, Linthicum, Maryland, United States; Center for Innovation, Linthicum, Maryland, United States

## Abstract

NASEM’s first recommendation to reform nursing home (NH) care is to deliver comprehensive, person-centered, equitable care that ensures residents’ health, quality of life, and safety, promotes autonomy, and manages risks. The NH Code of Federal Regulations requires person-centered care (PCC). However, while there are existing structures (i.e. MDS 3.0) to support care planning clinical needs, most NHs lack tools and procedures to systematically identify and provide care that aligns with resident goals, priorities, and preferences (GPPs). This is especially true for GPPs that reflect personhood and the human desire for autonomy. Our subcommittee aims to change the culture of care planning in NHs to integrate residents and families in co-developing holistic, person-centered care plans that centralize residents’ GPPs in ways that promote and support personhood. Our subcommittee will develop and test a) a standardized GPP care planning process that includes enhanced approaches to identification, documentation, communication, and implementation of resident GPPs; b) a repository of tools to support identification and documentation of resident GPPs; and c) training to prepare staff to understand and recognize resident GPPs. We will collaborate with other Moving Forward Committees to develop approaches to evaluating and measuring quality in care planning with inclusion of health information technology . When completed, the proposed work will provide evidence of successful and feasible quality improvement approaches to care planning resident GPPs that can augment existing federal requirements and can inform future policy changes or guidance to surveyors.

